# Supplementary material for: Late Eating Is Associated with Obesity, Inflammatory Markers and Circadian-Related Disturbances in School-Aged Children
Source: Nutrients. 2020 Sep 21;12(9):2881. doi: 10.3390/nu12092881 (PMC7551460; doi:10.3390/nu12092881)
Supplement: Supplementary file 1 [file nutrients-12-02881-s001.pdf]

Supplementary table 1. Differences in BMI and waist circumference between Late Dinner Eaters (LDE) and Early Dinner Eaters (EDE) separated by sex.

**Boys**	**LDE**		**EDE**		***P-value***
	Mean	SEM	Mean	SEM	
Age (years)*	10	0.1	10	0.1	0.065
BMI (kg/m^2)	19.2	0.4	19.2	0.4	0.988
BMI z-score	1.2	0.3	1.1	0.2	0.840
Waist circumference (cm)	66.5	1.2	66.1	1.1	0.814
Overweight/obesity (%)	16.4		16.4		0.578
**Girls**					
Age (years)*	10	0.1	10	0.1	0.903
BMI (kg/m^2)	19.8	0.3	18.1	0.3	**<0.001**
BMI z-score	1.5	0.2	0.6	0.2	**0.001**
Waist circumference (cm)	66.2	0.9	61.2	0.9	**<0.001**
Overweight/obesity (%)	23.0		10.0		**0.001**

Data were analyzed by ANCOVA adjusted by schools, age and academic year. *adjusted by schools and academic year. Significant differences are represented in boldfaces.

Supplementary table 2. Relationship of BMI z-score with intraindividual variation of dinner timing and the midpoint of food intake.

	BMI z-score		
	**Beta**	**SEM**	***P-value***
**Dinner phase deviation**	0.000	0.008	0.955
**Midpoint of food intake phase deviation**	- 0.009	0.005	0.089
**Interday phase change in dinner timing**	- 0.003	0.007	0.724
**Interday phase change in midpoint of intake**	0.006	0.006	0.323

Supplementary table 3. Differences in social jet lag of dinner timing and midpoint of food intake between boys and girls and across school grades.

	Social jet lag dinner timing (min)	Social jet lag midpoint of food intake (min)	P-value Social jet lag dinner timing	P-value Social jet lag midpoint of food intake
**Boys**	-0.4 (31.1)	39.0 (33.4)	0.500	0.521
**Girls**	1.5 (34.5)	41.2 (36.0)		
**Grade 1** (Age 8-9y)	6.4[a] (32.2)	45.0[a] (34.5)	**<0.001**	**<0.001**
**Grade 2** (Age 10-11y)	-14.2[b] (27.4)	23.2[b] (29.2)		
**Grade 3** (Age 11-12y)	6.0[a] (34.1)	48.4[a] (34.5)		

Mean (SD). Differences among grades (ages) assessed by ANOVA. Significant differences are represented in boldfaces. Different superindices indicate significant differences among grades.
